# Equal nonbreeding period survival in adults and juveniles of a long-distant migrant bird

**DOI:** 10.1002/ece3.984

**Published:** 2014-02-17

**Authors:** Martin U Grüebler, Fränzi Korner-Nievergelt, Beat Naef-Daenzer

**Affiliations:** 1 Swiss Ornithological InstituteSeerose 1, CH-6204, Sempach, Switzerland; 2 oikostat GmbH, Statistical Analysis and ConsultingAusserdorf 43, CH-6218, Ettiswil, Switzerland

**Keywords:** Avian demography, bird migration, *Hirundo rustica*, life-history stages, population ecology, postfledging survival

## Abstract

In migrant birds, survival estimates for the different life-history stages between fledging and first breeding are scarce. First-year survival is shown to be strongly reduced compared with annual survival of adult birds. However, it remains unclear whether the main bottleneck in juvenile long-distant migrants occurs in the postfledging period within the breeding ranges or en route. Quantifying survival rates during different life-history stages and during different periods of the migration cycle is crucial to understand forces driving the evolution of optimal life histories in migrant birds. Here, we estimate survival rates of adult and juvenile barn swallows (*Hirundo rustica*L.) in the breeding and nonbreeding areas using a population model integrating survival estimates in the breeding ranges based on a large radio-telemetry data set and published estimates of demographic parameters from large-scale population-monitoring projects across Switzerland. Input parameters included the country-wide population trend, annual productivity estimates of the double-brooded species, and year-to-year survival corrected for breeding dispersal. Juvenile survival in the 3-week postfledging period was low (*S* = 0.32; SE = 0.05), whereas in the rest of the annual cycle survival estimates of adults and juveniles were similarly high (*S* > 0.957). Thus, the postfledging period was the main survival bottleneck, revealing the striking result that nonbreeding period mortality (including migration) is not higher for juveniles than for adult birds. Therefore, focusing future research on sources of variation in postfledging mortality can provide new insights into determinants of population dynamics and life-history evolution of migrant birds.

## Introduction

Ecological factors determining variation in survival rates during different life-history stages are an important force driving the evolution of optimal life histories (Charlesworth 1994). Predicting population responses to environmental changes (such as climate change or habitat degradation) requires consideration of all life-history stages (Radchuk et al. 2013). Seasonal migrants visit geographically separated habitats with strongly varying environmental conditions. These may cause differential local survival rates (Schaub et al. 2011; Alves et al. 2013; Klaassen et al. 2014). Mortality en route is suggested to be particularly high (Newton 2006). Thus, understanding the mechanisms underlying the variation in annual survival and population changes requires identifying the bottlenecks in survival across life-history stages, in particular in the breeding and nonbreeding areas and during migration (Holmes 2007; Faaborg et al. 2010; Reid et al. 2011). During life-history transitions, such as fledging, survival rates are often strongly reduced, and to recognize such patterns is central for the understanding of variation in population dynamics (Low and Pärt 2009). However, while estimates of annual survival are available for many organisms including most bird species, survival rates have rarely been obtained for the different stages of the annual life cycle, even less for migrating organisms.

The migration cycle of long-distant migrant birds covers a large proportion of the nonbreeding part of their life, and the migration period is hypothesized to be a dangerous stage (Sillett and Holmes 2002; Newton 2006; Calvert et al. 2009; Klaassen et al. 2012). Thus, a major part of the annual mortality is considered to occur during migration. Potential causes include the intense metabolic demands (Åkesson and Hedenström 2007) and elevated predation risk (Lindström 1989; Sillett and Holmes 2002). Some studies also demonstrated increased migration mortality due to weather conditions, such as storm events (Newton 2007), extreme temperatures or strong precipitation (Møller 1989; Jones et al. 2004; Norman and Peach 2013). However, only few studies reported survival estimates for the critical nonbreeding life-history stages of migrant birds, as it is most challenging to disentangle the mortality in and outside of the breeding ranges. Moreover, studies conducted in the wintering habitats of migrant birds allowing quantification of nonbreeding survival and estimation of mortality during migration are still rare (Ketterson and Nolan 1982; Sillett and Holmes 2002).

First-year survival of migrant birds, that is, survival from fledging to the first breeding event, including the whole first migration cycle, is shown to be strongly reduced compared with adult breeding birds (Clark and Martin 2007; Tarof et al. 2011; Redmond and Murphy 2012), and its variation often contributes to population growth rate (Sæther and Bakke 2000). The first year involves distinct life-history stages such as the postfledging period, the first autumn and spring migration, and the first period in the wintering habitat. These stages most probably differ with respect to survival and to their impact on the over-all first-year survival (Robinson et al. 2004). However, little empirical work has been carried out to identify the crucial phase that contributes most to the pronounced difference in survival between first-year and older birds. In general, survival differences between adult and juvenile birds can develop in the breeding areas after fledging or, alternatively, in the nonbreeding areas including the migration journey. An increasing body of literature shows that in the nonbreeding period, young birds face elevated mortality risks (Owen and Black 1989; Menu et al. 2005; Newton 2006; Calvert et al. 2009; Guillemain et al. 2010) and that migration behavior of juveniles differs from that of adult birds (Thørup et al. 2003; Wiltschko and Wiltschko 2003). On the other hand, we have increasing evidence that postfledging survival in many migrant birds is low (Anders et al. 1997; Yackel Adams et al. 2006; Berkeley et al. 2007; Grüebler and Naef-Daenzer 2008a, 2010a). Thus, after the postfledging period, juvenile survival might not differ largely from that of adults. However, it is largely unknown to what extent the low first-year survival accrues from postfledging, migration or nonbreeding periods.

**Figure 1:**
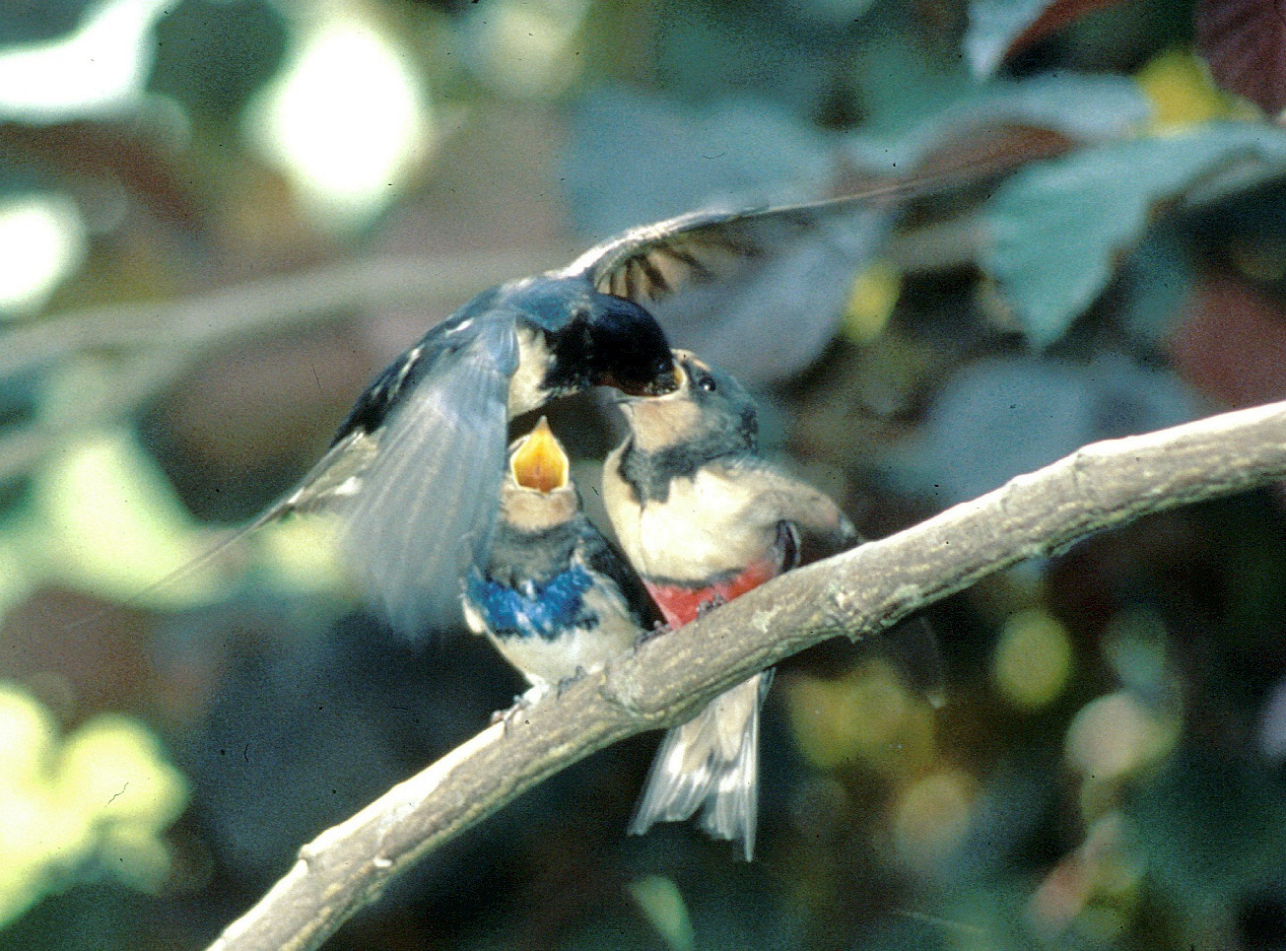
Adult barn swallow (*Hirundo rustica*L.) feeding color-marked and radio-tagged fledglings.

The aim of this study is to estimate mean survival rates of a long-distant migrant, the barn swallow (*Hirundo rustica* L.; Fig. 1), for the major age-and stage-related periods. We use a refined population model integrating the following information: (i) own radio-tracking data on postfledging juvenile survival and adult survival in the breeding ranges, and (ii) published estimates for dispersal-corrected annual adult survival, annual fecundity, and population trend. This approach allows for quantification of separate adult survival rates for the breeding and the nonbreeding areas, and estimation of unbiased juvenile postfledging and nonbreeding survival. The study gives new insights into the timing of demographic bottlenecks in migrant birds, the demographic importance of increased mortality during migration, and the main drivers of population dynamics.

## Materials and Methods

**Figure 2:**
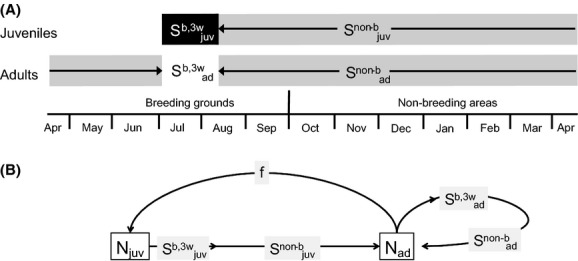
Illustration of the population model used to estimate age-and stage-specific survival of barn swallows. (A) Age-and stage-specific rates in the model as experienced within the life cycle of barn swallows, shown for adult birds and juveniles, respectively. (B) Age-and stage-specific rates as represented in the population model. The adult population in the year *t* + 1 was considered to be the sum of the surviving juveniles and adults from year *t*. Surviving juveniles were estimated by considering fecundity (reproduction rate *f*, leading to the number of juveniles of year *t*;*N*_*juv*_), postfledging juvenile survival (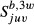), and juvenile nonbreeding survival (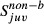). Surviving adults were estimated using adult survival in the breeding (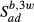) and in the nonbreeding areas (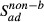). The focal parameter of the model was juvenile nonbreeding survival, whereas for all other parameters, estimates from own empirical data or from literature were available.

We combined demographic information on Swiss barn swallow populations from different sources to build a population model identifying survival estimates of adults and juveniles in the breeding grounds and in the nonbreeding areas (Fig. 2). The barn swallow is a long-distance migrant bird species, breeding in agricultural farms below 1200 m.a.s.l., arriving at the breeding grounds in April or May and leaving them again at end of September. Data sources included a radio-tracking study conducted in a single study area, providing new data on juvenile postfledging survival and adult survival in the breeding area, a country-wide population monitoring program providing population trend, and a large-scale volunteer-based barn swallow project in Switzerland (1997–2004) providing published results of mark–recapture and fecundity analyses (Table 1). Where nothing else is mentioned, analyses were carried out in R 2.15.2 (R Development Core Team 2012). The software WinBUGS was used via the R-interface “R2WinBUGS” (Sturtz et al. 2005).

**Table 1:** Sources of the input parameters of the model, number of study areas, and years covered.

Estimates	Study, number of study areas	Years covered	Reference
Population trend *λ*	Swiss Breeding Bird Index [barn swallow], 267 study areas across Switzerland	1997–2004	Zbinden et al. (2005)
Annual reproductive output *f*	Swiss Swallow Project, 13 study areas across Switzerland	1997–2004	Grüebler et al. (2010)
Annual adult survival *S*_*ad*_	Swiss Swallow Project, 8 study areas across Switzerland	1997–2004	Schaub and Von Hirschheydt (2009)
Adult survival (breeding sites) 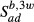	Wauwilermoos study area (Grüebler and Naef-Daenzer 2008a, 2010b)	2004	This study
Postfledging survival 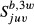	Wauwilermoos study area (Grüebler and Naef-Daenzer 2008a, 2010b)	2000, 2002–2004	This study

### Adult survival in the breeding grounds

In the breeding season 2004, we radio-tagged adult barn swallows during the nestling period at the Wauwilermoos study area in Switzerland using radio tags of own construction (Naef-Daenzer et al. 2005; Grüebler and Naef-Daenzer 2010b). For detailed methods on catching and radio tagging of adult barn swallows, and for description of the study area, see recent publications (Grüebler and Naef-Daenzer 2008a, 2010b). We tagged 11 males and 11 females and tracked them during 5–12 weeks. This resulted in 22 encounter histories with weekly encounters. As encounter probability was close to one for radio-tracked adult barn swallows during the breeding season, we used known fate survival models in the software package MARK 4.3 (White and Burnham 1999) to estimate weekly survival for adults during the breeding season. Our data confirm that adult breeding birds stay at the same farm throughout the breeding season and do not disperse to distant places between broods of the same year (Turner 2006; own unpubl. data), assuming that these survival estimates are close to the true survival. Two candidate models were tested: a model with and a model without sex-specific survival probability. For both models, constant survival over time was assumed.

### Postfledging survival

In the years 2000 and 2002–2004, postfledging juvenile survival was estimated in the same study area by radio tagging juvenile barn swallows just before fledging and subsequently locating them twice a day for the first 5 weeks from fledging. All radio tags (including battery and harness) had a mass of 650–750 mg, which represents 3.8–4.4% of the minimum fledgling mass (17 g). More methodological details about tagging and locating fledgling barn swallows and separate estimates of postfledging survival for first and second broods are given in recent publications (Grüebler and Naef-Daenzer 2008a, 2010a,b). Analyses showing no effect of radio tags on fledgling survival and quantifying the effect of characteristics of the tags on survival estimates in the same study on barn swallows are provided in Naef-Daenzer and Grüebler (2014). Here, we pooled encounter histories of the two broods to get average estimates of postfledging survival over the whole season.

In total, we radio-tagged 560 fledglings of 132 broods in the four study years (2000: 60 fledglings of 15 broods; 2002: 211 fledglings of 51 broods; 2003: 203 fledglings of 47 broods, 2004: 86 fledglings from 19 broods; single broods: 66 fledglings of 15 broods, first broods: 256 fledglings of 56 broods, second broods: 238 fledglings of 61 broods). The ratio of fledglings from single and double broods in the sample resembles the natural ratio in Switzerland, that is, *ca*. 80% double-brooded and 20% single-brooded pairs (Grüebler et al. 2010). Therefore, our survival estimates may be close to the Swiss average postfledging survival, even if survival differs between single, first, and second broods.

In contrast to breeding adults that regularly return to the nest, fledglings increase their range continuously and may remain undetected on some days during the 3-week period until they are detected again (Naef-Daenzer and Grüebler 2014). Therefore, detection probability of radio-tagged juveniles was less than one. To account for the detection probability, we used a Cormack–Jolly–Seber type of model to estimate daily survival and daily encounter probability (Lebreton et al. 1992). We allowed for fully time-dependent daily survival probabilities with independent estimates for each day (from fledging) within year. We included a normally distributed random family effect in the linear predictor for daily survival probability to control for the interdependency between fledglings of the same family. The logit function was used to link the linear predictor to survival probability. The logit of the encounter probability was linearly related to factors known to be associated with encounter probability from earlier survival analyses (Grüebler and Naef-Daenzer 2008a, 2010b). Following factors were included into the encounter model: age of the fledgling, duration of postfledging parental care (measured as the duration from fledging to the last feeding event observed in a family in days, Grüebler and Naef-Daenzer 2008b), an indicator whether the brood was a second brood or not, and, only for the second brood fledglings, the date of fledging. Detailed results showing that there is only a date effect on encounter probability in second broods but not in first broods are shown in Grüebler and Naef-Daenzer (2008a, 2010b). The linear predictor for encounter probability further contained normally distributed random year and family effects. The effect of age of the fledglings was allowed to differ between the years (random slope). The model was fitted to the data using Markov chain Monte Carlo (MCMC) simulations with the program WinBUGS (Lunn et al. 2009). Convergence of the Markov chains was graphically assessed and by the Brooks–Gelman–Rubin statistics (Brooks and Gelman 1998). Details of the model and model code are given in the Appendix S1.

From the daily survival probabilities averaged over the 4 years, we obtained the probability that an individual survived until day 21, that is, 3-week postfledging survival probability 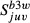, as the product of daily survival probabilities up to day 21. The errors of the daily survival probabilities were propagated to the estimate for the 3-week postfledging survival by calculating 3-week postfledging survival for all MCMC simulations of the daily survival probabilities resulting in 6000 values. The mean and 2.5% and 97.5% quantiles were used as estimate and 95% credible interval. The estimate of postfledging survival was restricted to a 3-week period from fledging because up to this time we never lost any fledgling and the fledglings never left the study area during the tracking hours. After day 25, this occurred regularly, suggesting that survival estimates for longer periods are biased by dispersal out of the study area.

### Population trend

The estimate for the population trend during the years 1997 to 2004, the period with data on fecundity and adult survival across Switzerland, was based on the Swiss Breeding Bird Index (BI) for barn swallows (Zbinden et al. 2005). The index represents a year-specific measure of population size relative to the reference year 2000. The population trend parameter *λ = N*_*ad,t*+1_/*N*_*ad,t*_ was estimated using a linear regression of the logarithm of the BI_*t*_/100 on year. For this regression, the index_*t*_ – values were weighted proportional to 1/se(Index_*t*_)^2^ to account for the uncertainties in the estimates for index_*t*_. The exponential of the slope parameter corresponds to the average multiplicative change in index_*t*_, that is, index_*t + 1*_/index_*t*_, and was used as an estimate for *λ*. A standard error for *λ* was obtained by simulating 1000 values from the posterior distribution of the slope parameter (function sim from the package arm, Gelman and Hill 2007). The mean and standard error of *λ* was used in the population model to describe what we know about population trend by a normal distribution: *λ* ∼ *Norm*(0.971, 0.017).

### Annual reproductive output

The estimate of mean annual reproductive output (i.e., the annual number of fledglings per breeding pair) was taken from a study investigating the factors affecting reproductive output in 13 study areas throughout Switzerland from 1997 to 2004, also including the Wauwilermoos study area (Grüebler et al. 2010), where the radio-tracking studies took place (Table 1). The mean number of fledglings (juveniles surviving the nestling period up to fledging) produced annually by a breeding pair was estimated to 6.12 ± 0.06 fledglings (mean ± SE). This information was transformed to a normal distribution *F* ∼ *Norm*(6.12, 0.06^2^), and *f* = *F*/2 was used as fecundity parameter (number of fledglings produced by one individual per year) in the population model.

### Annual adult survival

Estimates for adult year-to-year survival probabilities were taken from Schaub and Von Hirschheydt (2009) (Table 1). Their study was based on mark–recapture data of eight study areas across Switzerland, including also the Wauwilermoos study area. Adult survival is often underestimated, because breeding dispersal is common in many species (Schaub and Von Hirschheydt 2009; Pasinelli et al. 2011; Bötsch et al. 2012). Therefore, in population models estimating juvenile survival from known population parameters, an underestimation of adult year-to-year survival results in an overestimation of juvenile first-year survival. Schaub and Von Hirschheydt (2009) used a multistate model allowing individuals to disperse. Thus, the adult year-to-year survival estimates of their study were less biased than those of conventional mark–recapture studies, which do not differentiate between dispersal and survival. In particular, they showed that male and female annual survival did not differ for birds with high reproductive success, but females with low reproductive success dispersed more often than males, resulting in lower apparent survival rates for females when not controlled for dispersal. From the given adult survival estimates, we used the two values for second-year and older males with high reproductive success, as authors suggest that these estimates are only marginally biased by dispersal. These values were higher than previously published data on adult survival of barn swallows. We used the mean of the two estimates as an estimate of *S*_*ad*_. A standard error for this estimate was obtained using Monte Carlo simulation. To do so, we simulated 6000 random values from each of two beta distributions with mean and standard deviations equal to the estimates and standard errors of the two adult survival estimates. From these samples, we calculated 6000 pairwise means which together described the uncertainty in the estimate of *S*_*ad*_.

### Population model

We combined the demographic parameters from different sources assuming an age-structured Leslie matrix population model (Leslie 1945; Fig. 2). Estimating first-year survival of juvenile barn swallows using ringing–recapture data is very limited because recapture rates within study areas are below 4%, leading to survival estimates highly biased by natal dispersal. Thus, from the annual population trend, the annual adult survival, and the fecundity parameter, we derived first-year survival of the juveniles. Taking into account postfledging survival and adult survival in the breeding area allowed the estimation of nonbreeding survival of adults and juveniles (Fig. 2). The adult population in the year *t* + 1 (*N*_*ad,t* + 1_) was considered to be the sum of the surviving juveniles and adults from year *t*. Assuming that the number of fledglings of the year *t* (after surviving the nestling period, *N*_*juv,t*_) survives the first 3 weeks from fledging with the probability 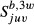 (postfledging survival) and thereafter to the first breeding season with the probability 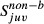 (nonbreeding survival), and that the number of adults of the year *t* (*N*_*ad,t*_) survives to the next breeding season with the probability *S*_*ad*_, the adult population is:


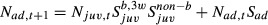
(1)

The number of juveniles in the year *t* (*N*_*juv,t*_) was assessed by the product of the adult population (*N*_*ad,t*_) and the reproduction rate *f*:


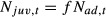
(2)

The population growth rate *λ* was defined as


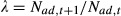
(3)

By inserting equations (2) and (3) into equation (1), juvenile nonbreeding survival (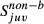) could be estimated by the equation


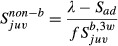
(4)

We used Monte Carlo simulations to obtain uncertainty estimates for 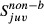. To do so, we applied equation (4) to each of 6000 sets of values (*λ*,*S*_*ad*_, *f* and 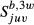) that were first drawn from the parameter-specific distributions reflecting the uncertainty in the specific parameter. As a result, we received 6000 values for 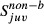 that reflected our knowledge about juvenile nonbreeding survival given our model assumptions and the information on the input parameters. The 2.5% and 97.5% quantiles of these 6000 values for 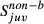 were used as lower and upper limit of the 95% credible interval.

To compare juvenile postfledging survival to adult survival in the breeding season, weekly adult survival was powered by three to obtain the 3-week survival of adults in the breeding area 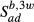. 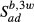 served to transform adult annual survival to adult nonbreeding survival (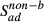) by the equation 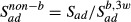. Annual juvenile survival was estimated as the product of the postfledging survival and the juvenile nonbreeding survival: 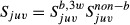.

The population model assumes that *S*_*ad*_ is an estimate for true adult survival, that is not confounded with dispersal. The estimate is taken from Schaub and Von Hirschheydt (2009) who largely took into account breeding dispersal. However, the estimate may still underestimate true survival, particularly when some of the adult males with high reproductive success do not return to the study area. We therefore performed a sensitivity analysis investigating the sensitivity of the juvenile nonbreeding survival estimate to a possible bias in the adult survival estimate. Detailed methods and results are presented in the Appendix S2. Similarly, as the estimate for adult breeding survival is based on a limited sample of radio-tracked adults of 1 year, this estimate might be biased and is expected to vary from year to year. We therefore performed a second sensitivity analysis investigating the sensitivity of the adult nonbreeding survival estimate to potential variation in the adult breeding survival estimate. Methods and results are also given in the Appendix S2.

## Results

### Adult survival in the breeding grounds

Model selection criteria favored the model with constant sex-independent adult survival (S_(.)_: QAICc = 24.28, np = 1; S_(sex)_: QAICc = 26.32, np = 2; *Δ*QAICc = 2.04). The estimate of weekly adult survival within the breeding season was *S* = 0.990 (SE = 0.007). Three-week survival was calculated to compare survival estimates in different life-cycle stages and amounted to 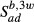 = 0.971 (SE = 0.020; Table 2). Assuming that adult barn swallows stay 23 weeks in the breeding ranges, total survival in the breeding area amounted to *S* = 0.798 (SE = 0.12).

**Table 2:** Population parameters and survival estimates, SE, and 95% credible intervals used and derived from the population model.

Parameter	Description	Time period (weeks)	Mean	SE	2.5%	97.5%
*λ*	Population trend	–	0.965	0.022	0.922	1.010
*f*	Annual reproductive output	–	3.060	0.030	3.001	3.119
*S*_*ad*_	Annual adult survival	52	0.475	0.020	0.436	0.515
*S*_*juv*_	Juvenile first-year survival	52	0.160	0.010	0.141	0.179
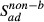	Adult nonbreeding survival	49	0.490	0.023	0.447	0.538
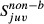	Juvenile nonbreeding survival	49	0.507	0.081	0.377	0.694
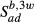	Adult survival breeding sites	3	0.971	0.020	0.922	0.996
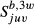	Juvenile postfledging survival	3	0.322	0.045	0.235	0.411
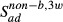	Adult nonbreeding survival	3	0.957	0.003	0.952	0.963
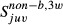	Juvenile nonbreeding survival	3	0.959	0.009	0.942	0.978

For comparability reasons, survival estimates are shown for different time periods (in weeks). Annual reproductive output per individual represents half of the annual output per pair; b, breeding grounds; non-b, nonbreeding ranges, including migration; 3 w, standardized to a 3-week period.

### Postfledging juvenile survival

**Figure 3:**
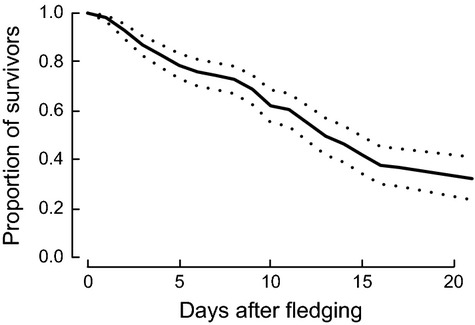
Proportion of surviving fledglings averaged over the four study years related to their age (in days after fledging). Dotted lines represent the credibility interval (an equivalent to the confidence interval). Postfledging survival 3 weeks after fledging (21 days) was used in the population model.

As expected from previous studies, postfledging survival probabilities changed with increasing age of the fledglings and differed between the 4 years of the telemetry study. The proportion of survivors in relation to the time since fledging is shown in Figure 3. Postfledging survival until 3 weeks after fledging was 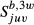 = 0.322 (SE = 0.045; Table 2). Details of parameter estimates of the postfledging survival analysis including factors affecting encounter probability and family random effects are presented in Appendix S1.

### Survival rates across the life cycle

The Swiss barn swallow population showed a negative average trend during the period 1997 to 2004. The population trend parameter *λ* = *N*_*ad t*+1_/*N*_*ad t*_ was *λ* = 0.965 (SE = 0.022; Table 2). As expected, estimates of annual survival rates of juveniles (first-year survival: *S*_*juv*_ = 0.160, SE = 0.010) were significantly lower compared with annual survival rates of adults (*S*_*ad*_ = 0.475, SE = 0.02; Table 2). Similarly, 3-week postfledging survival of juveniles was significantly lower than 3-week survival of adults in the breeding grounds (Table 2). In contrast, survival rates of juveniles during the nonbreeding period were similar to that of adults (49 weeks; 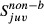 = 0.507, SE = 0.081; 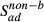 = 0.490, SE = 0.023; Table 2). This implies that the large difference between annual survival rates of adults and juveniles was caused by the low juvenile survival during the 3-week postfledging period. Moreover, juvenile survival probability during the postfledging period was significantly lower than juvenile survival during the nonbreeding period. Three-week adult survival showed no significant differences between breeding grounds and the nonbreeding areas (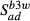 vs. 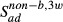; Table 2).

## Discussion

Prereproductive survival of juvenile birds defined as the survival from fledging to the first reproduction is known to be much lower than year-to-year survival of adults (Redmond and Murphy 2012; McKim-Louder et al. 2013). However, in migrant birds, it remains unclear to which particular life-history stage this pattern is related. Our results provide clear evidence that in barn swallows, the postfledging period is the main bottleneck, whereas during the rest of the year mean survival rates of juvenile and adult birds are similar. We therefore suggest that the mortality associated with the first migration and the stay at an unknown nonbreeding site is low compared with that just after fledging, and that nonbreeding period mortality is not higher for juveniles than for adult birds.

Recently, researchers postulated to increase the accuracy of estimates of productivity and survival used in population models, because model parameters often are biased due to imperfect estimation (Anders and Marshall 2005; Calvert et al. 2009; Faaborg et al. 2010; McKim-Louder et al. 2013). To our knowledge, this is the first study identifying the main bottleneck in survival for a long-distant migrant, integrating unbiased annual productivity estimates, year-to-year survival estimates largely corrected for breeding dispersal, and estimates of postfledging survival.

We show that the major part of overall mortality within barn swallow populations occurs in the breeding grounds during a very short time of the year, the postfledging period. This is in line with studies separately investigating postfledging survival: fledging is a life-history transition in which survival rates are strongly reduced (Anders et al. 1997; Naef-Daenzer et al. 2001; Grüebler and Naef-Daenzer 2008a; Low and Pärt 2009; Hovick et al. 2011; Reid et al. 2011). Factors operating in the short postfledging period therefore may be most important for long-term population dynamics and evolution of life histories, because they influence the ultimate reproductive success and the productivity of populations. Proximately, environmental conditions affecting body condition in the early life up to fledging are important determinants of postfledging survival (Grüebler and Naef-Daenzer 2008a, 2010a; Rivers et al. 2012) and carry over to body condition at the beginning of migration (Mitchell et al. 2011). Food availability and predation pressure can operate during both the nestling and the postfledging period, and they may represent crucial factors acting after fledging at two scales. First, individual nest site selection might be an important decision affecting individual breeding success also in species with low nestling mortality, because local food conditions and predation pressure result in differential postfledging survival (Berkeley et al. 2007). Second, changes in the breeding environment such as changes in large-scale predator populations or food availability might have strong effects on population dynamics by negatively affecting postfledging survival. Further evolutionary consequences develop, because postfledging survival also depends on the parental behavior after fledging (Grüebler and Naef-Daenzer 2010b; Naef-Daenzer et al. 2011). Parental time and energy constraints or parental strategies in the postfledging period may be important factors for life-history evolution, but remain widely unknown. Detailed investigation of variation in postfledging mortality may provide great new insight into evolutionary influences in life-history and parental care strategies among bird species (Martin 2002, 2004).

The fact that postfledging mortality is much larger than mortality during the rest of the nonbreeding period does not mean that there are no periods of increased mortality after leaving the breeding areas. However, our results suggest that survival over the whole nonbreeding period does not differ between adults and juveniles. Estimates of adult survival in the nonbreeding ranges (including migration journeys) tended to be lower than that in the breeding grounds. We may roughly estimate survival costs during migration by making two assumptions. First, we assume that survival in the nonbreeding home ranges is equal to that in the breeding ranges, which is suggested by recent studies (Sillett and Holmes 2002; Jones et al. 2004). Second, we assume that the period in the breeding and nonbreeding grounds is 23 weeks each, while the period en route is 6 weeks (3 weeks for each migration, Turner 2006; F. Liechti pers. comm.). Under these assumptions, 3-week survival during migration would amount to *S* = 0.86 compared with *S* = 0.97 in the breeding and nonbreeding home ranges. Survival over the whole periods would then roughly amount to 0.80 in the breeding and nonbreeding ranges and 0.75 during migration. However, to properly investigate this issue, separate empirical estimation of survival rates either en route and/or in the nonbreeding home ranges would be required.

Survival estimates vary temporally and spatially. For example, annual variation in first-year survival is influential to population growth rate (Robinson et al. 2004; Sim et al. 2011; Schaub et al. 2012) or conditions during migration are often associated with population dynamics affecting adult and juvenile survival (Stokke et al. 2005; Norman and Peach 2013). However, to what extent variation in postfledging survival is associated with population dynamics remains largely a “black box” of avian demography, only recently coming into the focus of ornithological research (Faaborg et al. 2010; Reid et al. 2011). Therefore, increasing the accuracy of age-and stage-specific survival estimates is necessary to enhance our understanding of population dynamics, reproductive trade-offs, and evolution of avian life histories.

## Supporting Information

Appendix S1 Modeling postfledging survival in Win-BUGS

Appendix S2 Sensitivity analyses
