# Supplementary material for: Equal nonbreeding period survival in adults and juveniles of a long-distant migrant bird
Source: Ecol Evol. 2014 Feb 17;4(6):756–65. doi: 10.1002/ece3.984 (PMC3967901; doi:10.1002/ece3.984)
Supplement: Appendix S1 — Modeling postfledging survival in Win-BUGS [file ece30004-0756-sd1.docx]

**Appendix SA1**Modelling post-fledging survival in WinBUGS

**1. Model description and settings of the Markov Chain Monte Carlo simulation**

To estimate daily post-fledging survival based on mark-recapture data of radio-tagged fledglings (the data can be downloaded from: http://datadryad.org/), we used a type of Cormack-Jolly-Seber model formulated as a state-space model. We modelled the encounter of individual *i* at day *t*, *y_it_*, as Bernoulli-distributed, *y_it_* ~Bernoulli(*p_it_z_it_*), where *p_it_* is the probability that individual *i* is re-encountered at day *t,* given it is alive and *z_it_* is a latent indicator variable of the state (0 = dead, 1 = alive) of the individual *i* at day *t*. For the latent state variable, we assumed another Bernoulli model: *z_it_*~Bernoulli(*φ_it_ z_it-1_*), where *φ_it_* is the survival probability of individual *i* from day *t*-1 to day *t*. We used the logit-link function to relate *φ_it_* and *p_it_* to their linear predictors. In the linear predictor for* φ_it_* we used a year-specific intercept and added a family-specific random effect. The random effects of the family were multiplied by duration since the last three intervals were longer than 1 day, i.e. from day 18 to 21, 22 to 28 and 29 to 38.

logit(*φ_it_*) = *b*^0^_year[_*_i_*_]_*_t_* + *b^f^*_family[_*_i_*_]_duration*_t_*

with *b^f^*_family_ ~ Norm(0, *_ _*_family_).

The logit of p was modelled linearly depending on the time since fledging, the duration of parental care, a binary indicator variable of second broods and the interaction between date and second brood (date only affected the detection probability of the second brood fledglings). We included a random year effect on the intercept and the slope for the time since fledging and we added a random family effect:

logit(*p_it_*) = *a*^0^_year[_*_i_*_]_ + *a*^1^_year[_*_i_*_]_day*_t_* + *a*^2^care*_i_* + *a*^3^secondbrood*_i_* + *a*^4^datesecondbrood*_i_* + *a^f^*_family[_*_i_*_]_

with *a*^0^_year_ ~ Norm(*a*^0mean^, *_a0_* _year_), *a*^1^_year_ ~ Norm(*a*^1mean^, *_a1_* _year_) and *a^f^*_family_ ~ Norm(0,  *_p_* _family_).

For the coefficients of the linear predictors we used uniform prior distributions Unif(-10, 10), and for the inverse of the between group variance of the random effects flat Gamma-distributions, Gamma(0.01, 0.01). Two chains of the length 80’000 were run, the first 20’000 were discarded and from the remaining 60’000 every 20^th^ value was used to describe the posterior distribution of the model parameters.

**2. Additional model results: encounter probabilities and random effects**

Model results of age-dependent post-fledging survival estimates are given in the main text (result section, subtitle: post-fledging survival; Fig. 3). In addition, factors affecting encounter probability and estimates of random family effects are presented here (Table S1).

***Encounter model:*** Encounter probability of fledglings significantly declined with time from fledging (slope of the regression line: parameter name: *a*^1mean^; intercept of the regression line: parameter name: *a*^0mean^; Table S1). Annual variation in radio-tag characteristics resulted in annually different slopes and intercepts of the decline (see Naef-Daenzer & Grüebler 2014). In our model, this variation was captured in the variance of the mean slope (parameter name: 1/(σ*_a_*_1year_)^2^) and the variance of the intercept (parameter name: 1/(σ*_a0_* _year_)^2^). As already known from earlier analyses (Grüebler & Naef-Daenzer 2008a, 2010b), the duration of post-fledging parental care showed a significant positive effect on encounter probability (parameter name: *a*^2^), whereas post-fledging encounter probability significantly declined with date in second broods (parameter name: *a*^4^; Table S1). When controlling for these two effects, encounter estimates of first and second broods showed no significant difference (parameter name: *a*^3^).

***Random family effects:*** The results of our model show that barn swallow families significantly differ in encounter probability (parameter name: 1/(σ*_p _*_family_)^2^) and survival probability (parameter name: *_ _*_family_)^2^), suggesting that post-fledging encounter and survival of fledglings of the same family are not independent (Table S1).

**Table S1.** Parameter estimates, standard errors and credibility intervals of factors affecting encounter probability and random family effects. R-hat = Brooks-Gelman-Rubin statistics for the assessment of convergence of the Markov chains; neff = number of effective sample size. Variable names in the BUGS-code (see below) and parameter names of the model equation (see above) are given.

| Bugscode | Parameter name | Estimate | SE | lower | upper | R-hat | neff |
| --- | --- | --- | --- | --- | --- | --- | --- |
| a[1] | *a*^2^ | 0.527 | 0.087 | 0.360 | 0.703 | 1.001 | 6000 |
| a[2] | *a*^3^ | 0.051 | 0.187 | -0.320 | 0.414 | 1.001 | 3900 |
| a[3] | *a*^4^ | 0.447 | 0.144 | 0.162 | 0.727 | 1.002 | 1800 |
| mp | *a*^0mean^ | 2.007 | 0.493 | 1.112 | 2.935 | 1.001 | 6000 |
| msd | *a*^1mean^ | -0.148 | 0.108 | -0.345 | 0.072 | 1.005 | 6000 |
| tauicp | 1/(σ*_a0_* _year_)^2^ | 4.070 | 4.811 | 0.238 | 14.921 | 1.001 | 6000 |
| tauslopedayp | 1/(σ*_a_*_1year_)^2^ | 62.434 | 52.295 | 4.439 | 199.802 | 1.001 | 6000 |
| taufameffp | 1/(σ*_p _*_family_)^2^ | 1.706 | 0.378 | 1.088 | 2.558 | 1.001 | 6000 |
| taufameffphi | 1*_ _*_family_)^2^ | 2.096 | 0.710 | 1.125 | 3.797 | 1.001 | 6000 |
| deviance |  | 4546.212 | 51.948 | 4448.000 | 4650.000 | 1.013 | 470 |

**3. R- and Bugs-code of the survival model**

# Cormack-Jolly-Seber model with random effects to

# estimate post-fledling survival

#-----------------------------------------------------------------------------------

# load packages

library(R2WinBUGS)

#-------------------------------------------------------------------------------

# data

bugs.data <- dget("data.postfledling.survival.txt")

str(bugs.data)

#List of 11

# $ CH encounter histories for 560 individuals over 21 days after fledging

# $ nind number of individuals

# $ n.occasions number of days

# $ care.z duration of parental cares (z-transformed), individual covariate

# $ jahr year index

# $ d.z date for second broods (z-transformed), zero for single or first broods (interaction brood type x date)

# $ zweitbrut indicator of second brood

# $ duration duration of time interval (the last encounter occasions were longer than one day)

# $ day.c day since fledging (z-transformed)

# $ familie family index

# $ nfam number of families

#-------------------------------------------------------------------------------

# Model parameters:

# phi daily survival probability

# p daily encounter probability

# alive true state variable

# icphi year and day specific intercept of the linear predictor for daily survival probability

# icp year-specific intercept for encounter probability

# rfameffphi random family effects in the linear predictor for phi

# slopedayp year-specific slope for the effect of day on encounter probability

# a model coefficients in the linear predictor for p

# rfameffp random family effects in the linear predictor for p

# mp      mean of the intercept in the encounter model

# msd    mean of the slope for day in the encounter model

# tauicp inverse of between-year variance in the intercept for p

# tauslopedayp inverse of the between-year variance in the slope for day on p

# taufameffp inverse of the between-family variance in the logit of p

# taufameffphi inverse of the between-family variance in the logit of phi

#-------------------------------------------------------------------------------

# The model:

sink("cjs.txt")

cat("

model {

# Priors and constraints

for(i in 1:nind){

for (t in 1:(n.occasions-1)){

logit(phi[i,t]) <- icphi[jahr[i], t] + rfameffphi[familie[i]]*duration[t]

logit(p[i,t]) <- icp[jahr[i]] + slopedayp[jahr[i]]*day.c[t+1] + a[1]*care.z[i] +a[2]*zweitbrut[i] + a[3]*d.z[i] + rfameffp[familie[i]]

}

}

# random effects

for(j in 1:4){

icp[j]~dnorm(mp, tauicp)

slopedayp[j]~dnorm(msd, tauslopedayp)

}

for(f in 1:nfam){

rfameffp[f]~dnorm(0, taufameffp)

rfameffphi[f]~dnorm(0, taufameffphi)

}

# priors

for(i in 1:3){

a[i]~dunif(-10, 10)

}

for(t in 1:(n.occasions-1)){

for(j in 1:4){

icphi[j,t]~dunif(-10, 10)

}

}

tauicp ~dgamma(0.01, 0.01)

tauslopedayp ~dgamma(0.01, 0.01)

taufameffp ~dgamma(0.01, 0.01)

taufameffphi ~dgamma(0.01, 0.01)

mp ~dunif(-10, 10)

msd ~dunif(-10, 10)

# Define the likelihood

for (i in 1:nind){

# Ensures that individuals enter the sample with probability 1

alive[i,1] <- 1

for (t in 2:n.occasions){

# State process

alive[i,t] ~ dbern(mu1[i,t])

mu1[i,t] <- phi[i,t-1] * alive[i,t-1]

# Observation process

CH[i,t] ~ dbern(mu2[i,t])

mu2[i,t] <- p[i,t-1] * alive[i,t]

} # t

} # i

# derived parameters

for(t in 1:(n.occasions-1)){

for(j in 1:4){

logit(phidayt[j,t]) <- icphi[j,t]

}

phidaymean[t] <- mean(phidayt[1:4,t])

survdayt[t] <- prod(phidaymean[1:t])

}

}

",fill=TRUE)

sink()

#--------------------------------------------------------------------------------

# initial values

# Function, which creates a matrix of initial values for matrix "alive"

ch.init <- function(ch, first, possible.states, uncertain.state){

ch.iv <- ch

for (i in 1:dim(ch)[1]){ch.iv[i,1:first[i]] <- NA}

u <- which(ch.iv==uncertain.state)

for (i in 1:length(u)){ch.iv[u[i]] <- sample(possible.states,1)}

return(ch.iv)

}

inits <- function(){list(alive = ch.init(bugs.data$CH, rep(1, bugs.data$nind), c(0,1), 0),

tauicp=runif(1, 0.1, 1), tauslopedayp =runif(1, 0.1, 1), taufameffp =runif(1, 0.1, 1), taufameffphi=runif(1, 0.1, 1),

icphi=matrix(runif(4*(ncol(bugs.data$CH)-1), -1, 1), nrow=4), a=runif(3, -1, 1))}

#--------------------------------------------------------------------------------

# Define parameters to be monitored

parameters <- c("survdayt", "a", "mp", "tauicp", "tauslopedayp", "taufameffp", "taufameffphi")

#--------------------------------------------------------------------------------

# MCMC settings

niter <- 80000

nthin <- 20

nburn <- 20000

nchains <- 2

bugs.dir <- "c:/Programme/WinBUGS14"

#--------------------------------------------------------------------------------

# Do the MCMC stuff calling WinBUGS from R

cjs <- bugs(bugs.data, inits, parameters, "cjs.txt", n.chains = nchains,

n.thin = nthin, n.iter = niter, n.burnin = nburn, debug = T, bugs.directory = bugs.dir, working.directory = getwd())

#--------------------------------------------------------------------------
