# Supplementary material for: Equal nonbreeding period survival in adults and juveniles of a long-distant migrant bird
Source: Ecol Evol. 2014 Feb 17;4(6):756–65. doi: 10.1002/ece3.984 (PMC3967901; doi:10.1002/ece3.984)
Supplement: Appendix S2 — Sensitivity analyses [file ece30004-0756-sd2.docx]

**Appendix SA2** Sensitivity analyses

**1. Sensitivity of the juvenile non-breeding survival estimate to a potential bias in the annual adult survival estimate**

**Methods**

Dispersal of adult barn swallows might be larger than quantified by Schaub & Von Hirschheydt (2009).Therefore, we investigated the sensitivity of the juvenile non-breeding survival estimate (*S^non-b^_juv_*) to a possible bias in the adult survival estimate (*S_ad_*). We varied the proportion of adults that may have dispersed from the study area of Schaub & Von Hirschheydt (2009), and thus the bias in the adult survival estimate, from 0% to 10%. Then, we replaced adult annual survival probability *S_ad_* in our study by varying true adult survival estimates that were corrected for the proportions of potential dispersers.

**Results**

The estimates of juvenile non-breeding survival were fairly robust against violation of the assumption that the annual adult survival estimates used in the model were unbiased from dispersal (Fig. S1). If we assume an (unrealistic large) proportion of 10% additionally emigrating adults, our estimate for juvenile non-breeding survival decreased to *S^non-b^_juv_* = 0.452 (CrI = 0.332 – 0.625), whereas the corresponding adult survival increased to *S^non-b^_ad_* = 0.544 (CrI = 0.496 – 0.597). Up to 6% additional emigration is possible without reaching a significant difference between adult and juvenile non-breeding survival (Fig. S1).

**Figure S1.** Estimates for adult and juvenile non-breeding survival in relation to potential biases in the estimate of annual adult survival derived from non-accounted breeding dispersal. Proportions of 0 to 0.1 additionally dispersing adults (x-axis) were assumed.


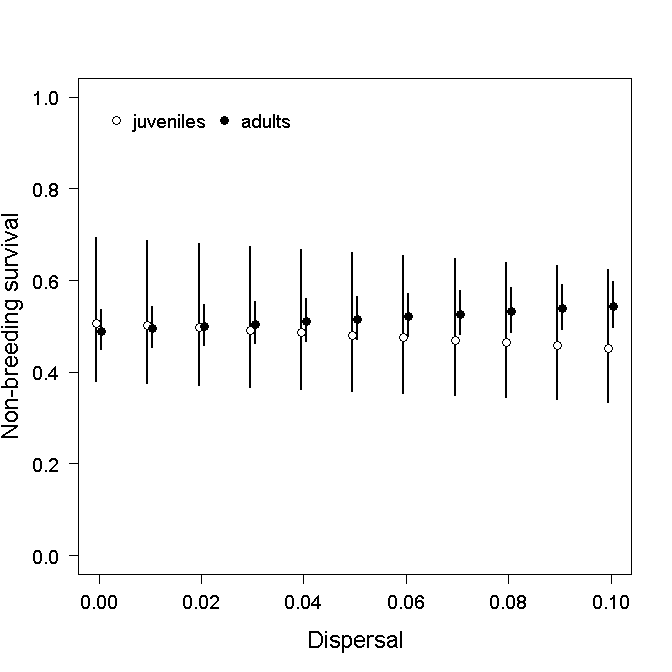


**2. Sensivity of the adult non-breeding survival estimate to variation in the adult breeding survival estimate**

**Methods**

The estimate of adult survival during the breeding period was based only on a limited sample of radio-tracked birds from one single year. Thus, estimates of adult breeding survival used in the population model can considerably differ from the true mean value over many breeding seasons. Consequently, we investigated the sensitivity of the adult non-breeding survival estimate (*S^non-b^_ad_*) to a possible bias in the adult breeding survival estimate (*S^b,3w^_ad_*). We varied the weekly adult breeding survival estimate in our model from 0.980 to 0.999 (equivalent to a three-week survival of 0.941 to 0.997, or equivalent to the survival over the whole breeding period of 0.63 to 0.98).

**Results**

The estimates of adult non-breeding survival changed only slightly in the investigated range of adult breeding survival estimates (Fig. S2). If we assume a large positive or negative bias in the adult breeding survival estimate (maximal survival deviation of 0.18 over the whole breeding season), the estimates for adult non-breeding survival varied between *S^non-b^_ad_* = 0.505 (CrI = 0.459 – 0.554) and *S^non-b^_ad_* = 0.477 (CrI = 0.436 – 0.521). In this range of potential biases, we clearly found no evidence for differences between adult and juvenile non-breeding survival (Fig. S2). Considering that the year of adult radio-tracking (2004) was a year with favourable weather and low nestling survival, we would expect an overestimation of mean adult breeding survival rather than an underestimation. However, lower adult breeding survival even resulted in a smaller difference between adult and juvenile non-breeding survival.

**Figure S2.** Estimates for adult (filled circles) and juvenile (open circles) non-breeding survival in relation to potential biases in the estimate of adult breeding survival. Values of weekly adult breeding survival of 0.980 to 0.999 (x-axis) were assumed.

**
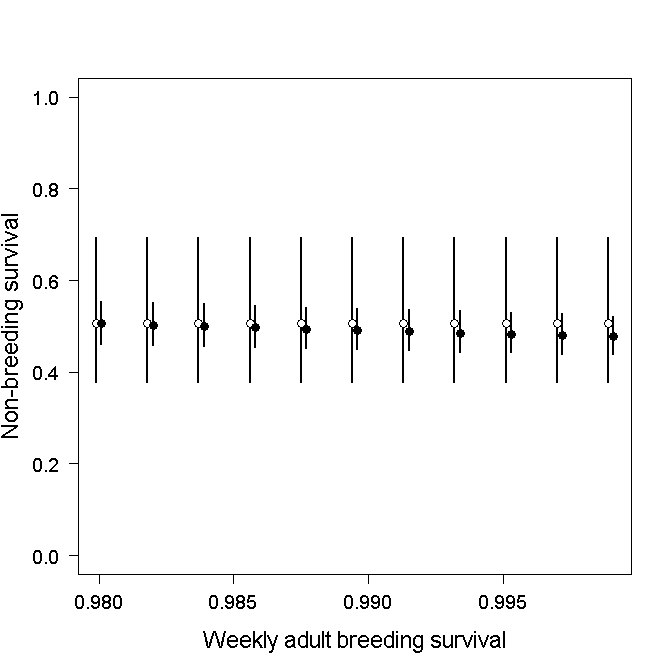
**
